# Simultaneous Optimization of Vehicle Arrival Time and Signal Timings within a Connected Vehicle Environment

**DOI:** 10.3390/s20010191

**Published:** 2019-12-29

**Authors:** Wei Wu, Ling Huang, Ronghua Du

**Affiliations:** 1 Key Laboratory of Highway Engineering of Ministry of Education, Changsha University of Science & Technology, Changsha 410114, Hunan, China; 2 School of Civil Engineering and Architecture, Changsha University of Science & Technology, Changsha 410114, Hunan, China; 3 College of Automotive and Mechanical Engineering, Changsha University of Science & Technology, Changsha 410114, Hunan, China

**Keywords:** traffic signal control, speed guidance, vehicle arrival time, connected vehicle

## Abstract

Most existing signal timing plans are optimized given vehicles’ arrival time (i.e., the time for the upcoming vehicles to arrive at the stop line) as exogenous input. In this paper, based on the connected vehicle (CV) technique, vehicles can be regarded as moving sensors, and their arrival time can be dynamically adjusted by speed guidance according to the current signal status and traffic conditions. Therefore, an integrated traffic control model is proposed in this study to optimize vehicle arrival time (or travel speed) and signal timing simultaneously. “Speed guidance model at a red light” and “speed guidance model at a green light” are presented to model the influences between travel speed and signal timing. Then, the methods to model the vehicle arrival time, vehicle delay, and number of stops are proposed. The total delay, which includes the control delay, queuing delay, and signal delay, is used as the objective of the proposed model. The decision variables consist of vehicle arrival time, starting time of green, and duration of green for each phase. The sliding time window is adopted to dynamically tackle the problems. Compared with the results optimized by the classical actuated signal control method and the fixed-time-based speed guidance model, the proposed model can significantly decrease travel delays as well as improve the flexibility and mobility of traffic control. The sensitivity analysis with the communication distance, the market penetration of connected vehicles, and the compliance rate of speed guidance further demonstrates the potential of the proposed model to be applied in various traffic conditions.

## 1. Introduction

Traffic signal control is one of the most effective methods to alleviate traffic congestion [1,2]. The existing signal control strategies can be divided into three categories, namely fixed time, actuated, and adaptive [3]. Fixed-time-based traffic control utilizes the historical traffic volume at an intersection to calculate the parameters for signal timing (e.g., cycle length, green time split, and offset) [4,5]. Fixed-time-based traffic control may not be able to adapt to demand fluctuation caused by the stochastic nature of traffic arrivals, which may degrade its benefits of signal control. In order to tackle the real-time demand fluctuation, actuated and adaptive traffic control methods have been proposed. By collecting real-time traffic arrivals from preinstalled sensors (e.g., loops, microwaves, and videos), actuated control methods can adjust their signal timing on the basis of a few simple rules, that is, green extension and minimum and maximum green duration constraints [6,7,8,9]. For adaptive control methods, they predict traffic arrivals first by utilizing the data collected from preinstalled sensors and then optimize the predefined metrics (e.g., minimize delays, maximize throughputs) [10,11,12]. Whether actuated control or adaptive control is involved, their signal plans are optimized based on the data collected from preinstalled sensors. However, these sensors (e.g., loops, microwaves, and videos) are only installed at a few fixed points near the intersection and can only collect traffic data at those limited points. Indeed, intersection signal controllers are not able to grasp the accurate and complete information of traffic arrivals based on these limited sensors, which may result in the inefficiency of the traffic signal control. Furthermore, the preinstalled sensors (e.g., loops, microwaves, and videos) can only monitor vehicles as they are approaching an intersection; they are not able to influence or change traffic arrivals based on the current signal timings to achieve better performance. 

With the development of wireless communication technology such as radio-frequency identification (RFID), dedicated short-range communication (DSRC), and 5G (the fifth generation of cellular network technology), a connected vehicle (CV) technique has emerged and is being employed in recent years [13,14,15,16,17,18,19]. By equipping vehicles with an on-board unit (OBU), the connected vehicles are able to communicate with surrounding vehicles and infrastructures (e.g., signal controllers) in real time [3,20,21,22], and these connected vehicles can be regarded as moving sensors. Therefore, first, the trajectory data collected from the connected vehicle such as real-time speed, acceleration, and location can be transferred to the signal controller, then signal plans can be optimized with more precision [23,24]. Second, with connected vehicles, some critical metrics such as queue length can be detected or estimated [25,26,27,28], which can be incorporated to reflect the performance and influence the optimization of signal plans. This may further improve the efficiency of traffic control.

Most existing studies merely focus on how to optimize signal plans based on the trajectory data collected from the connected vehicle [3,20,29,30]. In particular, based on the trajectory data, vehicles’ arrival times (i.e., the time for vehicles to arrive at the stop line) around the intersection can be estimated with more precision, then signal plans can be optimized by taking these vehicles’ arrival times as exogenous input. However, based on the two-way communication between vehicles and the signal controller in a connected vehicle environment, a speed guidance or speed advisory can be deployed. For example, Wan et al. [31] proposed a speed advisory system (SAS) for reducing idling and improving energy efficiency at a red light. Ramezani and Benekohal [32] claimed that the accuracy of data and the effectiveness of speed guidance could be enhanced based on a connected vehicle technique, and they developed an optimization program to determine advisory speeds for connected vehicles in work zones. Tajalli and Hajbabaie [33] regulated the movement of vehicles to achieve a “smoother” flow of traffic by harmonizing the speed of connected vehicles.

By using a speed guidance or speed advisory, the arrival time of connected vehicles can be dynamically optimized by adjusting their travel speed according to the current signal status and traffic conditions [34,35,36]. This means that not only signal plans can be adjusted to adapt to traffic arrivals but also traffic arrivals can be adjusted likewise to adapt to the signal plans in a connected vehicle environment. Specifically, Tang et al. [35] for the first time introduced driver’s bounded rationality into a speed guidance model. The impacts of driver’s bounded rationality on fuel consumption and emissions were studied. Tang et al. [36] further introduced a speed guidance strategy into a car-following model. Wu et al. [34] proposed a speed guidance model for a fixed-time-based signal controller. However, signal timing plans were not simultaneously optimized along with the speed guidance strategy in those studies. In particular, the existing studies focused mainly on modelling speed guidance strategies without optimizing signal timing plans at the same time, especially when many connected vehicles were coming from conflicting movements (e.g., through vehicles from the east and through vehicles from the south).

This paper fills the mentioned gap and contributes to the literature in several ways. First, this study examines the dynamic interaction between signal timing and vehicle arrival time (or travel speed). This has been rarely modelled in the literature. In particular, two speed guidance strategies associated with dynamic signal timing plans and three types of vehicle delays (i.e., control delay, queuing delay, and signal delay) are modelled. Second, by using the Standard North American NEMA (National Electrical Manufacturers Association) Dual-Ring structure, the proposed model is among the first to simultaneously optimize intersection signal timings and recommended travel speed for connected vehicles coming from conflicting movements at an intersection in a united framework. Specifically, the proposed model calculates the optimal signal timing to decrease the total delay for all approaching connected vehicles as well as optimizes the travel speed for each connected vehicle at the same time. It should be noted that both signal timing and travel speed adjustments may improve travel efficiency for one vehicle; however, it remains a great challenge to simultaneously allocate green time to each phase and calculate the optimal travel speed for all approaching connected vehicles, especially when these connected vehicles are coming from conflicting movements.

The remainder of this paper is organized as follows. Section 2 starts with a simple example of a connected vehicle to clear the intersection with either signal timing or speed adjustments. Notations and descriptions are also provided in Section 2. Section 3 models the problem, and the speed guidance model, delay model, and objective function are proposed to simultaneously optimize vehicle arrival time and signal timing plans. Numerical examples are presented in Section 4 to illustrate the model and analysis. Section 5 concludes the paper.

## 2. Problem Description and Parameter Definition

**Figure 1:**
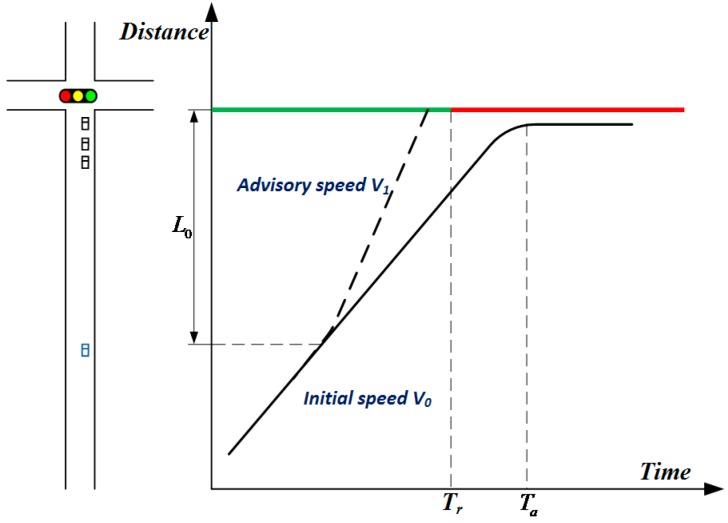
Signal timing and vehicle arrival.

Taking the end of the green light as an example, as shown in Figure 1, if the vehicle proceeds without speed adjustment, it reaches the intersection at $T_{a}$ and stops because the signal turns red at $T_{r}$. If the vehicle needs to pass the intersection without stopping, the common practice is to extend the duration of the green light for $T_{a}-T_{r}$ seconds, which means the existing methods need to obtain the vehicle arrival time as inputs to optimize the signal timing and extend the green light. However, vehicles could also clear the intersection without stopping by other means. For example, guiding the driving speed from $V_{0}$ to $V_{1}$ can help the vehicle arrive at the intersection during green time without recalculating the signal timing. This means that either optimizing the signal timing or adjusting the arriving time (or speed) can make the vehicle clear the intersection without stopping. However, most existing studies focus mainly on signal timing optimization or speed guidance. In this paper, both signal timing and speed are assumed to be optimized simultaneously. Note that when these two variables are optimized for each vehicle, the complexity and optimization space of the model are greatly increased. The research problem in this paper can be specified as follows: how to obtain the optimal vehicle arrival time and the signal timing for all approaching vehicles around the intersection to ensure the maximum performance of the intersection.

The main notations and their definitions and descriptions are presented in Table 1.

## 3. Optimization Model

### 3.1. Speed Guidance Associated with Dynamic Signal Timing Plans

When modelling speed guidance, it is assumed that the travel paths for each connected vehicle are preplanned. Therefore, through two-way communication between the signal controller and connected vehicles, the signal controller can acquire the vehicle’s turning decision (i.e., left turn, through, or right turn) in advance. Furthermore, vehicles shift to their chosen lane in advance when approaching an intersection according to their turning decisions, thus the negative impacts of lane changing were not incorporated into this research. In this context, in order to guide the vehicles to reach the intersection at the best time, this paper proposes two speed guidance models and establishes the mathematical relationship between the optimal driving speed and the starting time of the green light. Note that the starting times of the green light for each of the traffic movements are also variables and must be optimized (discussed in Section 3.5). According to different signal timings and traffic conditions, different speed guidance models can be established. There are mainly two models as follows:

Speed guidance model at a red light. When the vehicle’s requesting phase is yellow or red, or it is green but the queue has not dissipated, the proposed speed guidance model is called the “red-light guidance model”.

Speed guidance model at a green light. When the vehicle’s requesting phase is green and the queue has dissipated, the proposed speed guidance model is called the “green-light guidance model”.

The biggest difference between the red-light guidance and green-light guidance models is that the queue is not dissipated when the red-light speed guidance is implemented. In order to determine the vehicle’s optimal speed, we defined a “reference point” for each vehicle and assumed that the travel speed for this vehicle travelling from the current location to its “reference point” was constant. Under the red-light speed guidance, the reference point should take the queue length into account, whereas under the green-light guidance, because the queue has dissipated, the reference point for speed guidance is therefore at the stop line.

#### 3.1.1. Speed Guidance Model at a Red Light

The strategy of the red-light speed guidance is to ensure that when a vehicle reaches the end of the queue (caused by the red light), the shock wave is just transmitted to the end of the queue, that is, the vehicle at the end of the queue starts to move. In this context, this vehicle can smoothly clear the intersection following the shock wave. Therefore, the model for the red-light speed guidance can be written as follows:

(1) $$V_{t}(i,p,j)=\frac{L_{d}(i,p,j)-\sum_{j_{1}=1}^{j-1}L_{s}(i,p,j_{1})}{t(i,p,k)+\sum_{j_{1}=1}^{j-1}L_{s}(i,p,j_{1})/V_{s}-t_{n}}$$

In the above equation, the numerator indicates the distance between the vehicle and the stop line minus the road space occupied by all the preceding vehicles (queue length), and the denominator indicates the time duration between the time that the last vehicle in the queue begins to move and the current time. Note that the suggested speed (i.e., $V_{t}(i,p,j)$) and the vehicle’s arrival time (i.e., t(i,p,k)) are two variables to be optimized simultaneously.

#### 3.1.2. Speed Guidance Model at a Green Light

**Figure 2:**
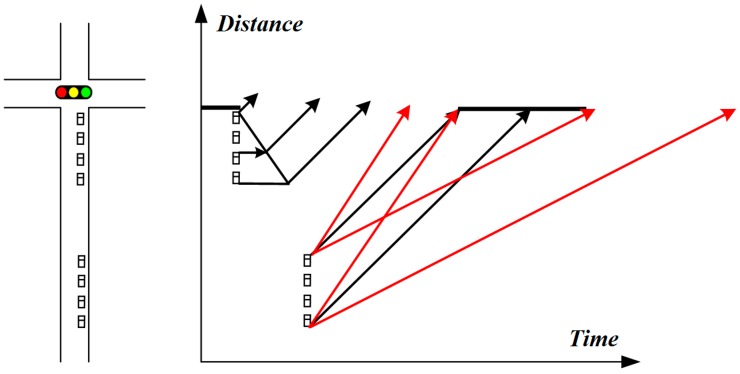
Speed guidance at a green light.

The effectiveness of the speed guidance model at a green light is presented in Figure 2. Note that the red line in Figure 2 is the trajectory of the guided vehicle, which means it can pass through the intersection without stopping by increasing the vehicle speed in the current cycle or by decreasing the vehicle speed and then passing through in the next cycle. Therefore, the speed guidance model at a green light can be specified as follows:

(2) $$V_{t}=L_{d}(i,p,j)/\{\sum_{j_{1}=1}^{j-1}L_{s}(i,p,j)/V_{a}(i,p,j)\cdot \phi (i,p,j,k)+[t(i,p,k+1)-t_{n}+\sum_{j_{1}=1}^{j-1}L_{s}(i,p,j)/V_{a}(i,p,j)]\cdot \phi (i,p,j,k+1)\}.$$

In the above equation, ϕ is a binary variable, which indicates whether the vehicle passes through the intersection in the current cycle or not. The optimization model in this paper only considers two cycles at a time, that is, vehicles in the entrance lane should pass through the intersection in the current cycle or in the next cycle, in other words as follows:

(3) ϕ(i,p,j,k)+ϕ(i,p,j,k+1)=1

Whether in the model of the red-light speed guidance or the green-light speed guidance, the suggested driving speed for a vehicle after guidance must have the following:

(4) $$V_{g}(i,p,j)=\{\begin{array}{c} V_{t}(i,p,j),\\ V_{\max},\\ V_{\min}, \end{array}\begin{array}{c} V_{\min}\leq V_{t}\leq V_{\max}\\ V_{t}(i,p,j)>V_{\max}\\ V_{t}(i,p,j)<V_{\min} \end{array}.$$

Equation (4) illustrates that the suggested speed must be constrained within the upper and lower bounds, namely $V_{\max}$ and $V_{\min}$.

### 3.2. Vehicle Arrival Time 

#### 3.2.1. Vehicle Arrival Time Model under the Red-Light Guidance

If $V_{g}(i,p,j)=V_{t}(i,p,j)$, it means that the vehicle can approach the intersection at an ideal speed. When it travels to the end of the queue, the last queued vehicle starts to move. Then, this vehicle follows the vehicle at the end of the queue to clear the intersection without stopping. In this case, the arrival time of the vehicle can be estimated as follows:

(5) $$T(i,p,j)=t(i,p,k)+\frac{\sum_{j_{1}=1}^{j-1}L_{s}(i,p,j_{1})}{V_{s}}+\frac{\sum_{j_{1}=1}^{j-1}L_{s}(i,p,j_{1})}{V_{a}(i,p,j_{1})}.$$

Equation (5) demonstrates that the arrival time of a vehicle is equal to the time of its preceding vehicle passing the stop line.

If $V_{g}(i,p,j)=V_{\max}$, it means that even if the vehicle travels at the maximum speed, it cannot catch up with the vehicle at the maximum queue point, that is, the queue has completely dissipated when the vehicle travels near the intersection, and its arrival time can be estimated by the following:

(6) $$T(i,p,j)=t_{n}+L_{d}(i,p,j)/V_{\max}.$$

Equation (6) demonstrates that the arrival time of a vehicle is equal to the current time plus the duration of traveling to the stop line at the highest speed.

If $V_{g}(i,p,j)=V_{\min}$, it means that even if the vehicle travels at the lowest speed, it will also move to the maximum queuing point and then stop. In this case, the arrival time of the vehicle can also be calculated by Equation (5), which means that the arrival time of the vehicle is equal to the time of its preceding vehicle passing through the stop line. However, in this case, the vehicle will stop because it arrives at the maximum queuing point before the queue is dissipated.

#### 3.2.2. Vehicle Arrival Time Model under the Green-Light Guidance

In the case of the green-light guidance, if $V_{g}(i,p,j)=V_{t}(i,p,j)$, it means that whether the vehicle passes through the intersection in the current cycle or in the next cycle, it can approach the intersection at an ideal speed without stopping. In this scenario, if the vehicle passes through the intersection within the rest of the green time in the current cycle, the arrival time of the vehicle can also be calculated by the Equation (5), that is, following the preceding vehicle to pass through the intersection. If the vehicle passes the green light in the next cycle (please refer to Figure 2), the arrival time of the vehicle can be estimated as follows:

(7) $$T(i,p,j)=t(i,p,k+1)+\frac{\sum_{j_{1}=1}^{j-1}L_{s}(i,p,j_{1})}{V_{s}}+\frac{\sum_{j_{1}=1}^{j-1}L_{s}(i,p,j_{1})}{V_{a}(i,p,j_{1})}.$$

Equation (7) demonstrates that the arrival time is equal to the time of the vehicle smoothly passing the stop line following the preceding vehicle in the next cycle.

If $V_{g}(i,p,j)=V_{\max}$, it means that even if the vehicle travels at the maximum speed, it cannot catch up with the preceding vehicle when passing through the intersection in the current cycle. In this context, its arrival time can be estimated by Equation (6). If $V_{g}(i,p,j)=V_{\min}$, it means that even if the vehicle runs at the lowest speed, it will pass through the intersection in the next cycle; then, the arrival time of the vehicle can be estimated by Equation (7).

### 3.3. Prediction of Vehicle Delay

Vehicle delay includes control delay, queuing delay, and signal delay, where control delay is caused by the change in speed due to speed guidance, queuing delay is caused by the queue, and signal delay is caused by the red light [1].

#### 3.3.1. Control Delay

In the case of the red-light guidance, where the point of the maximum queue length is taken as the reference point for the speed guidance, the control delay caused by the speed change can be calculated by the following:

(8) $$d_{c}(i,p,j)=\frac{L_{d}(i,p,j)-\sum_{j_{1}=1}^{j-1}L_{s}(i,p,j_{1})}{V_{g}(i,p,j_{1})}-\frac{L_{d}(i,p,j)-\sum_{j_{1}=1}^{j-1}L_{s}(i,p,j_{1})}{V_{a}(i,p,j_{1})}.$$

In the case of the green-light guidance, if the vehicle speed is guided with the stop line as the reference point, the control delay caused by the speed change can be specified as follows:

(9) $$d_{c}(i,p,j)=L_{d}(i,p,j)/V_{g}(i,p,j)-L_{d}(i,p,j)/V_{a}(i,p,j).$$

#### 3.3.2. Queuing Delay and Signal Delay

**Figure 3:**
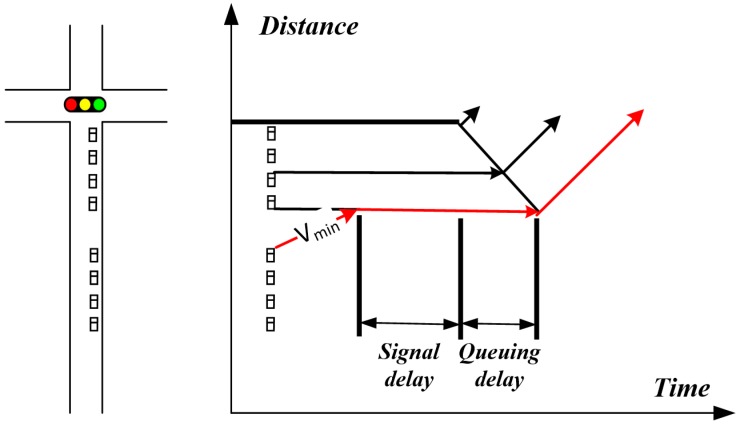
Queuing delay and signal delay caused by traffic control.

If $V_{g}(i,p,j)=V_{t}(i,p,j)$ or $V_{g}(i,p,j)=V_{\max}$, vehicles can smoothly pass the intersection without stopping, and the queuing delay and signal delay are zero. If $V_{g}(i,p,j)=V_{\min}$, under the guidance of a red light, queuing delay and signal delay are shown in Figure 3.

In this case, the signal delay and queuing delay can be written as follows:

(10) $$d_{s}(i,p,j)=t(i,p,k)-t_{n}-\frac{L_{d}(i,p,j)-\sum_{j_{1}=1}^{j-1}L_{s}(i,p,j_{1})}{V_{\min}},$$

(11) $$d_{q}(i,p,j)=\sum_{j_{1}=1}^{j-1}L_{s}(i,p,j_{1})/V_{s}.$$

In the case of the green-light guidance, the queuing delay and signal delay are similar, except that the starting point of the green light in Equation(10) (i.e., t(i,p,k)) should be replaced by t(i,p,k+1), which means that when $V_{g}(i,p,j)=V_{\min}$ under the guidance of a green light, this vehicle will pass through the intersection in the next cycle.

### 3.4. Prediction of Number of Stops

The number of stops is another important metrics to measure the effectiveness of traffic control. In this paper, the number of stops was reduced by adopting a speed guidance to adjust the arrival of traffic flow. Indeed, if $V_{g}(i,p,j)\geq V_{\min}$ in the proposed model, vehicles will pass through the intersection without stopping; if the guided speed is equal to the minimum speed limit, the vehicle will stop, in other words as follows:

(12) $$s(i,p,j)=\{\begin{array}{c} 0,\\ 1, \end{array}\begin{array}{c} V_{g}(i,p,j)\geq V_{\min}\\ V_{g}(i,p,j)=V_{\min} \end{array}.$$

### 3.5. Intersection Signal Control Model

**Figure 4:**
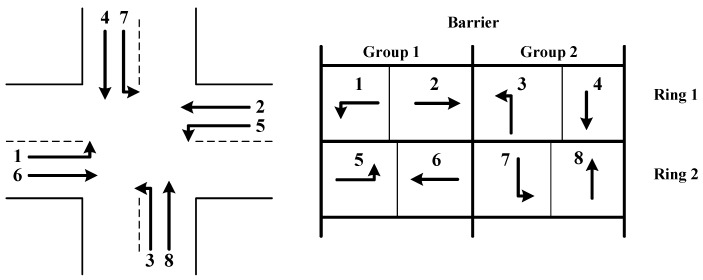
NEMA dual-ring structure for traffic signal control.

The intersection signal control model in this paper adopted the Standard North American NEMA Dual-Ring structure, as shown in Figure 4. The left diagram shows the basic shape of the intersection and the distribution of phases in each entrance lane. The right diagram shows the phases contained in each ring, where the first ring contains phases 1, 2, 3, and 4, and the second ring contains phases 5, 6, 7, and 8. All phases are divided into two groups: the first group includes phases 1, 2, 5, and 6, and the second group includes phases 3, 4, 7, and 8. The phases of the second group can only be operated until all phases in the first group finish. Note that based on the NEMA structure, phase skipping and phase insertion are not allowed in this study.

Similar to Head et al. [37] and He et al. [30], the dual-ring structure can be modelled according to the time sequence of phase execution, as shown in the following formula.

(13) t(i,1,1)=0

(14) t(i,5,1)=0

(15) t(i,2,k)=t(i,1,k)+v(i,1,k)

(16) t(i,6,k)=t(i,5,k)+v(i,5,k)

(17) t(i,3,k)=t(i,2,k)+v(i,2,k)

(18) t(i,3,k)=t(i,6,k)+v(i,6,k)

(19) t(i,7,k)=t(i,2,k)+v(i,2,k)

(20) t(i,7,k)=t(i,6,k)+v(i,6,k)

(21) t(i,4,k)=t(i,3,k)+v(i,3,k)

(22) t(i,8,k)=t(i,7,k)+v(i,7,k)

(23) t(i,1,k+1)=t(i,4,k)+v(i,4,k)

(24) t(i,1,k+1)=t(i,8,k)+v(i,8,k)

(25) t(i,5,k+1)=t(i,4,k)+v(i,4,k)

(26) t(i,5,k+1)=t(i,8,k)+v(i,8,k)

In the above, phases 1 and 5 are taken as starting points of optimization. The starting time of the green light of phase 2 (i.e., t(i,2,k)), is equal to the starting time of the green light of phase 1 (i.e., t(i,1,k)), plus the time of duration of phase 1 (i.e., v(i,1,k)). Note that the phase duration is equal to the sum of the duration of the green light and the green interval. Furthermore, the duration of the green light must be constrained within the upper and lower bounds, in other words as follows:

(27) $$g_{\min}\leq g\leq g_{\max}.$$

### 3.6. Objective Function

The objective function used in the proposed model was to minimize the total delay of all approaching vehicles around the intersection, which can be specified as follows:

$$\min\sum_{\left(i,p,j\right)}d(i,p,j),$$

where d(i,p,j) is the total delay of vehicle *j* from phase *p* at intersection *i*, which consisted of its control delay, queuing delay, and signal delay, in other words as follows:

(28) $$d(i,p,j)=d_{c}(i,p,j)+d_{q}(i,p,j)+d_{s}(i,p,j).$$

The control variables are the following:

Starting time of green light: t(i,p,k)

Green light’s duration: g(i,p,k)

Cycle selection parameters: ϕ(i,p,j,k)

Vehicle’s arrival time: T(i,p,j)

In the above control variables, the starting time of the green light (i.e., t(i,p,k)) is optimized for all the signal phases of an intersection. t(i,p,k) is mainly restricted by the constraints in Section 3.5 (i.e., intersection signal control model). The vehicle’s arrival time (i.e., T(i,p,j)) is decided by the vehicle’s speed. T(i,p,j) is mainly restricted by the constraints in Section 3.1 (i.e., speed guidance associated with dynamic signal timing plans). Note that the starting time of the green light and the vehicle’s arrival time are both control variables that are required to be optimized simultaneously to decrease the intersection delays.

### 3.7. Optimization Methods

**Figure 5:**
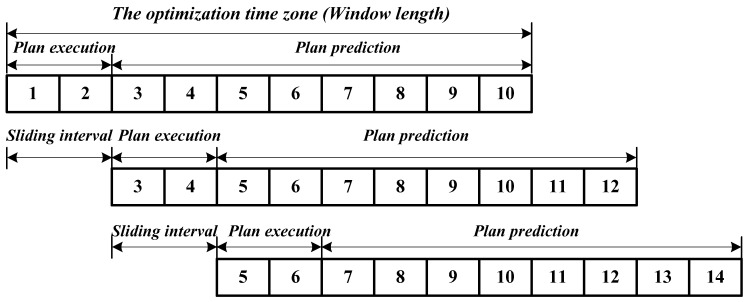
Illustration of the sliding time window method.

A sliding time window was used to dynamically optimize the arrival time of vehicles and the starting and duration of the green light of each phase. The implementation of the sliding time window is shown in Figure 5.

As shown in Figure 5, by using the sliding time window, signal timing and the vehicle’s speed are optimized in the optimization time zone (i.e., window length). The sliding interval is always identical with the plan execution time. The time duration of the window length is always longer than the sliding interval. This means that the outcomes after each optimization are not implemented for the whole window length. After a sliding interval (or plan execution time), a new optimization for a next window length is activated.

### 3.8. Optimization Procedures

In this paper, the optimization was based on VISSIM 5.3 simulation software (Planung Transport Verkehr AG, Karlsruhe, Germany) and the C++ programming environment in the Microsoft Visual Studio 2010 platform (Redmond, WA, USA). The COM interface was used to connect VISSIM and the C++ programming environment. The NEMA signal control module was used as the signal control execution module in the VISSIM simulation. With the sliding time window, the dynamic results of the signal control program optimized in the C++ program were transmitted to the NEMA control module for execution. In order to guide the vehicle speed, it was necessary to use the C++ program to modify the “desired speed” of the vehicle in VISSIM. The optimization mainly includes the following steps.

Step 1. Simulation initialization. Build the network model for the intersection in VISSIM software, input traffic flow and initial basic parameters into the NEMA control module.

Step 2. Simulation runs. Using C++ and the COM interface to connect VISSIM software and run the simulation. If the simulation runs to the starting point of a sliding time window, step 3 is executed.

Step 3. Recording the position and the speed of each vehicle in the entrance lane of the intersection, and recording the signal status of the current phase, including the light color (default 1 in VISSIM software is red light, 3 is green light, 4 is yellow light) and the time duration that the current signal has been executed.

Step 4. The model is optimized and solved to generate vehicle speed and signal timing scheme. In C++, all possible signal control schemes that meet all constraints in the current cycle and the next cycle are circulated, mainly including the parameters of the green light’s starting time and of the green light’s duration. The optimal guiding speed of the vehicle under all possible signal control schemes is calculated, and then the total delay of the vehicle under this guiding speed is calculated. Eventually, the signal control scheme and the speed guidance scheme with the minimum total delay of the vehicle are selected as the optimal scheme to be executed for the next sliding optimization step.

Step 5. Implementing the speed guidance scheme and the signal timing scheme. Note that the implementation of the signal control scheme is achieved by modifying the “Detection” attribute of the NEMA signal control module in the VISSIM–COM interface through the C++ program. The implementation of the speed guidance scheme is achieved by modifying the “Desired Speed” attribute in the VISSIM–COM interface through the C++ program. When step 5 is completed, go to step 2.

## 4. Model Verification

### 4.1. Experimental Design

In order to verify the effectiveness of the model proposed in this paper, based on VISSIM microsimulation software, a basic intersection consisting of two approaches (with two lanes at each approach) was used for the simulation. The saturation flow for each lane was 1900 pcu/h. For each entrance lane, the travel demand was 830 vehicles pcu/h for the benchmark case (i.e., the degree of saturation equals 1.0). Furthermore, we varied the travel demand for the performance analysis. For example, if the degree of saturation was λ, λ≥0, then the travel demand was 830λ. The method proposed in this paper was compared with the classical actuated signal control method and the speed guidance with fixed-time-based traffic signal control. The parameters used in each method are as follows.

Case 1. Actuated signal control method. In this method, a loop detector with a length of 18 m was placed at each entrance lane. The duration of the yellow light of each phase was set to 3 s and the all-red time was set to 2 s. Gap-out time of each phase was set to 3 s. The minimum duration of the green light for each phase was set to 5 s and the maximum duration of the green light was set to 35 s.

Case 2. Speed guidance with fixed-time-based traffic signal control proposed by Wu et al. [34]. In Case 2, the cycle time was 80 s. The green duration for each phase was 35 s. The duration of yellow and the all-red times were all identical with those in Case 1.

The proposed method. The length of the time window was set to 10 s. The duration of the yellow light, the duration of the all-red time, and the minimum and maximum duration of the green light were all identical with those in Case 1. The minimum speed limit was 20 km/h and the maximum speed limit was 60 km/h. The communication distance was set to 400 m, that is, when the distance between a connected vehicle and the stop line was less than 400 m, the signal controller was aware of this vehicle’s approach and optimized the suggested speed for this vehicle. Later, the communication distance was varied for the sensitivity analysis.

**Figure 6:**
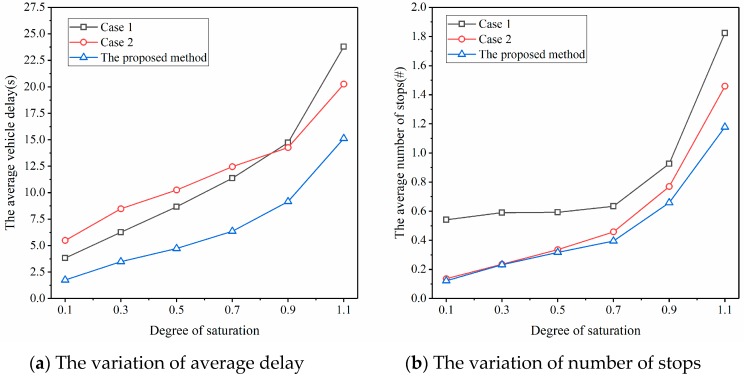
Comparison of average delay and average number of stops at different traffic saturation degrees.

In order to eliminate the impacts of the length of the entrance lane on the simulation results, the length of the entrance lane was set to 2000 m in the simulation. Each simulation ran for 2700 s (45 min), and the first 900 s were the pre-heating period of the simulation. The metrics including average delay and average number of stops were collected in the half-hour period between 900 and 2700 s. The simulation of each set of parameters used 20 different random seeds, and the average performance (i.e., average delay and average number of stops) under these different random seeds was used for comparison and analysis. The simulation results are shown in Figure 6.

A few observations can be made from Figure 6. First, with the increase in the degree of saturation, the average vehicle delay and the average number of stops of Case 1, Case 2, and the proposed method increased accordingly. However, whether in low saturation or high saturation, if we compare the proposed method with Case 1 (i.e., actuated signal control method), the average delay and the average number of stops are greatly reduced. The reduction range of the average vehicle delay is between 37.8% and 54.0%, and that of the number of stops is between 29.0% and 77.5%, which proves that the proposed method can effectively reduce the delays and number of stops and thus improve the efficiency of the intersection. This is exactly the advantage of the simultaneous optimization of vehicle arrival and signal timing proposed in this paper, which can allocate the green light and arrange vehicle arrivals in a more reasonable way, thereby improving the operation efficiency.

Second, if we compare the proposed method with Case 2 (i.e., speed guidance with fixed-time-based traffic signal control), the results clearly show that the proposed method outperforms Case 2 in terms of average vehicle delay. With respect to the number of stops, when the degree of saturation is relatively low (e.g., no greater than 0.5), the two methods provide almost the same performance. This means that speed guidance is extremely effective at low saturation, even when the signal timing is fixed. This result is similar to that in Wu et al. [34]. As the degree of saturation increases, and by simultaneously optimizing vehicle arrival and signal timing, the proposed method dominates Case 2 in terms of the number of stops.

Third, Case 2 always provides a smaller number of stops when compared with Case 1. This further verifies the effectiveness of speed guidance in decreasing the number of stops. With respect to the average vehicle delay, when the degree of saturation is less than 0.9, Case 1 with the actuated signal control provided a smaller vehicle delay. This occurs because actuated signal control allocates green time in a more reasonable way when the degree of saturation is low. However, at a high degree of saturation (e.g., when the degree of saturation is 1.1), the average vehicle delay in Case 2 with speed guidance is even smaller. This is the case because, when traffic volume is extremely heavy, actuated signal control only produces limited benefits, whereas speed guidance reduces the number of delays for vehicles.

### 4.2. Sensitivity Analysis 

#### 4.2.1. Sensitivity Analysis of the Communication Distance

**Figure 7:**
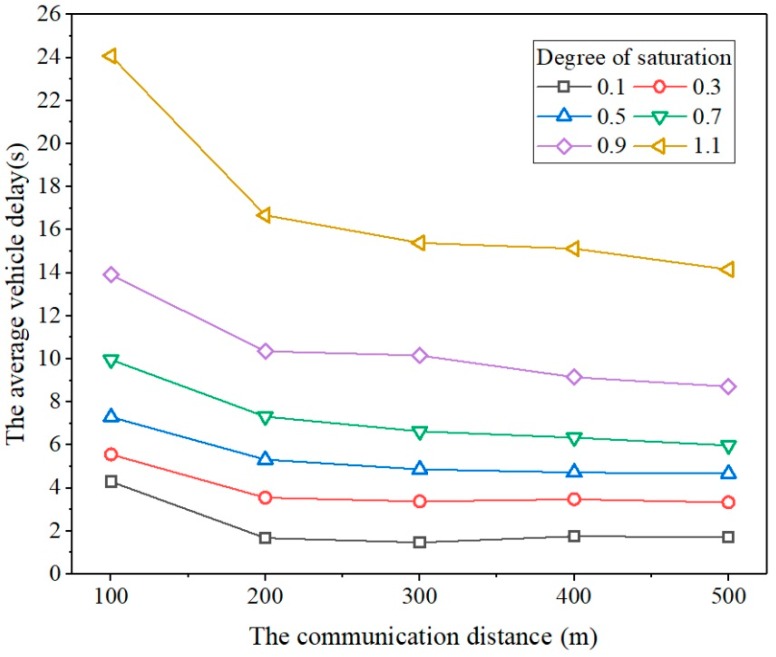
Comparison of average delay at different communication distances.

Communication distance is critical to the success of the proposed model. The signal controller can only acquire the vehicles’ arrival information within the communication distance. In addition, the speed guidance can only be conducted in the range of the communication distance. Therefore, the performance of the proposed model under different communication distances had to be investigated. By varying the communication distance from 100 to 500 m and varying the degree of saturation from 0.1 to 1.1, we obtained the simulation results that are shown in Figure 7.

In Figure 7, it is clearly shown that at a given degree of saturation, the average delay decreases with respect to the communication distance, that is, a larger communication distance provides a smaller average delay. This result is as expected. Furthermore, at a given degree of saturation, the average delay decreases obviously when the communication distance varies from 100 to 200 m. When the communication distance varies from 200 to 500 m, the decrements of average delay are not significant.

#### 4.2.2. Sensitivity Analysis of the Market Penetration of Connected Vehicles

When promoting two-way communication between vehicles and vehicles/infrastructures, it is inevitable that the number of connected vehicles will vary from fewer to more. The market penetration of connected vehicles will reach 100% over a long period of time. In the United States, it may take around 25 years for the connected vehicle’s occupancy rate to reach 100% after the new car is installed in the factory [3]. Therefore, it is necessary to discuss the effectiveness of the traffic control method proposed in this paper when connected vehicles and regular vehicles are mixed on the roads. Therefore, the impacts of the variation in the CV penetration on the proposed method were studied.

**Figure 8:**
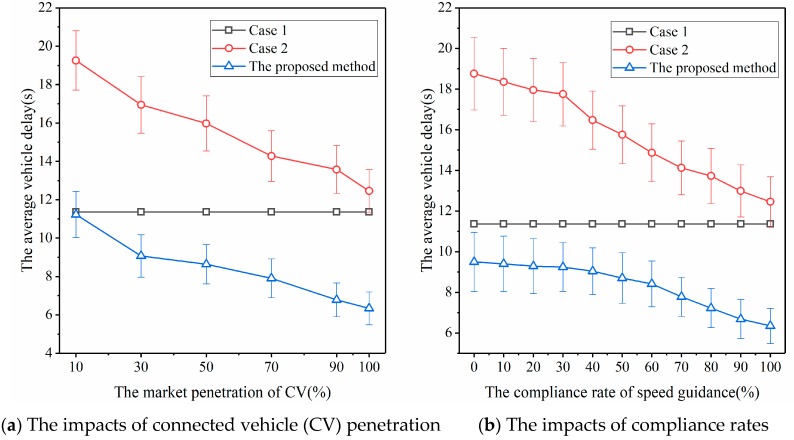
The variation of average delay under different connected vehicle (CV) penetration and compliance rates.

We fixed the degree of saturation to 0.7 and varied the market penetration of connected vehicles from 10% to 100%. Figure 8a shows the variation of average delays of all vehicles including connected vehicles and regular vehicles at the intersection for the three cases.

Figure 8a shows that as the market penetration of connected vehicles increases, the average vehicle delay for Case 1 (i.e., actuated signal control) stays the same because the actuated signal timing plan is loop-detector-based and does not depend on connected vehicles. For Case 2 and the proposed method, the average vehicle delay decreases with the increase of the market penetration of connected vehicles. In particular, by using the proposed method, as the market penetration of connected vehicles increases from 10% to 100%, the reduction of average vehicle delay is about 43.5%. Furthermore, when the CV penetration is very small, for instance 10%, in terms of vehicle delays, the performance of the proposed method can be better or even worse according to different traffic arrival patterns (or different random seeds; note that different random seeds will result in different traffic arrival patterns). With respect to the average vehicle delay, when CV penetration is 10%, the performance of the proposed method is almost identical to that of the actuated signal control method. This is the case because, when the CV penetration is small, the signal controller can only obtain information from fewer vehicles on the road, and speed guidance is difficult due to the obstruction between vehicles, which leads to poor efficiency. However, when the CV penetration reaches 30%, the efficiency of the traffic control method proposed in this paper is better than that of the actuated signal control method.

#### 4.2.3. Sensitivity Analysis of the Compliance Rate of Speed Guidance

One may consider that, in a real road traffic environment, not all drivers follow the speed advice from the traffic control system. This means that some drivers may not drive at the recommended speed. Therefore, the effectiveness of the traffic control model proposed in this paper had to be analyzed in this scenario. We fixed the degree of saturation to 0.7 and varied the compliance rate of speed guidance from 0% to 100%. The variation of the average vehicle delay for the three cases is shown in Figure 8b.

Figure 8b shows that as the driver’s compliance rate of speed guidance increases, the average vehicle delay decreases for Case 1 and the proposed method (i.e., cases with speed guidance). For Case 2 without speed guidance, the average delay stays the same. For the proposed method, from a compliance rate of 0% to 100%, the reduction of average vehicle delay is 33.2%, which can be considered as the effectiveness of vehicle speed guidance for the proposed method. In particular, when the compliance rate is 0%, which means all drivers do not follow the recommended speed, all the benefits are generated by signal control. In this extreme case, the proposed method can still reduce the average vehicle delay by about 16.4% compared with the vehicle delay by actuated signal control method. This further verifies that by using the trajectory data from connected vehicles, the proposed traffic control method can better allocate space–time resources and improve traffic efficiency compared with the actuated signal control method.

## 5. Conclusions

In this paper, an integrated traffic control model was established to optimize the vehicle arrival time and signal timing simultaneously, which overcomes the disadvantages of most existing traffic signal control models, which only passively adapt to vehicle arrivals. Based on the two-way communication between vehicle and signal controller, a speed guidance model, a vehicle arrival model, and a prediction model of delays and number of stops were proposed. The objective function was to minimize the average vehicle delay for all approaching vehicles. A sliding time window technique was used for dynamic optimization. VISSIM simulation results show that the proposed method can outperform both the classical actuated signal control method and the fixed-time-based speed guidance model. In particular, compared with the actuated signal control model, the proposed method can significantly reduce vehicle delays by about 37.8%–54.0% and the number of stops by about 29.0%–77.5%. A sensitivity analysis of the communication distance, the market penetration of connected vehicles, and the compliance rate of speed guidance further shows the effectiveness of the proposed model. Specifically, the proposed model can obtain similar or even better benefits compared with the classical actuated signal control when the CV penetration or the compliance rate of speed guidance is low. This verifies the advantages of the proposed traffic control model that simultaneously optimizes the vehicle arrival time and signal timing.

In order to highlight the effectiveness of the proposed traffic control model, this paper only used the trajectory data that could be obtained based on two-way communication. In fact, other sources of data such as a loop detector or video can also be collected. Further studies should be conducted to make use of each data source and improve the benefits of traffic control by data fusion methods, especially when the market penetration of connected vehicles is low. The case study used in this paper only analyzes a basic signalized intersection; more intensive performance testing of the proposed model in large-scale road networks while considering multiple road users such as pedestrians and cyclists may also be investigated in a future study.

## Figures and Tables

**Table 1:** Notations and descriptions.

Notations	Descriptions
(i,p,j)	Vehicle *j* from phase *p* at intersection *i*
$V_{\max}$	The maximum speed limit
$V_{\min}$	The minimum speed limit
$g_{\max}$	The maximum green time duration for a phase
$g_{\min}$	The minimum green time duration for a phase
$V_{s}$	The speed of traffic wave spreads
$V_{g}(i,p,j)$	The advisory speed for vehicle *j* from phase *p* at intersection *i*
$V_{a}(i,p,j)$	The regular travel speed for vehicle *j* from phase *p* at intersection *i*
$V_{t}(i,p,j)$	The optimum travel speed for vehicle *j* from phase *p* at intersection *i*
T(i,p,j)	The time for vehicle *j* from phase *p* at intersection *i* to arrive at the stop line
$T_{c}(i,p,j)$	The time length for vehicle *j* from phase *p* at intersection *i* to clear the intersection
$L_{d}(i,p,j)$	The distance between the stop line and vehicle *j* from phase *p* at intersection *i*
$L_{s}(i,p,j)$	The vehicle length of vehicle *j* from phase *p* at intersection *i*
d(i,p,j)	The total delay of vehicle *j* from phase *p* at intersection *i*
$d_{c}(i,p,j)$	The control delay of vehicle *j* from phase *p* at intersection *i*
$d_{q}(i,p,j)$	The queuing delay of vehicle *j* from phase *p* at intersection *i*
$d_{s}(i,p,j)$	The signal delay of vehicle *j* from phase *p* at intersection *i*
ϕ(i,p,j,k)	The binary variable, ϕ(i,p,j,k)=1, denotes vehicle *j* from phase *p* at intersection *i* will traverse the intersection in cycle *k*, zero otherwise
$t_{n}$	The current time
t(i,p,k)	The starting time of green light for phase *p* at intersection *i* in cycle *k*
g(i,p,k)	The time duration of green light for phase *p* at intersection *i* in cycle *k*
v(i,p,k)	The time duration of phase *p* at intersection *i* in cycle *k*, which is equal to the summation of the duration of green light and green interval of phase *p*

## References

[1] Mirchandani P., Head L. (2001). A real-time traffic signal control system: Architecture, algorithms, and analysis. Transp. Res. Part C Emerg. Technol..

[2] Lo H.K. (1999). A novel traffic signal control formulation. Transp. Res. Part A Policy Pract..

[3] Feng Y., Head K.L., Khoshmagham S., Zamanipour M. (2015). A real-time adaptive signal control in a connected vehicle environment. Transp. Res. Part C Emerg. Technol..

[4] Serafini P., Ukovich W. (1989). A mathematical model for the fixed-time traffic control problem. Eur. J. Oper. Res..

[5] Muralidharan A., Pedarsani R., Varaiya P. (2015). Analysis of fixed-time control. Transp. Res. Part B Methodol..

[6] Hao P., Wu G., Boriboonsomsin K., Barth M.J. (2018). Eco-approach and departure (EAD) application for actuated signals in real-world traffic. IEEE Trans. Intell. Transp. Syst..

[7] Shiri M.S., Maleki H.R. (2017). Maximum Green Time Settings for Traffic-Actuated Signal Control at Isolated Intersections Using Fuzzy Logic. Int. J. Fuzzy Syst..

[8] Liu Z., Bie Y. (2015). Comparison of hook-turn scheme with U-turn scheme based on actuated traffic control algorithm. Transp. A Transp. Sci..

[9] Wu W., Liu Y., Liu W., Zhang F., Rey D., Dixit V. (2019). An integrated approach for optimizing left-turn forbiddance decisions at multiple intersections. Transp. B Transp. Dyn..

[10] Mannion P., Duggan J., Howley E. (2016). An experimental review of reinforcement learning algorithms for adaptive traffic signal control. Autonomic Road Transport Support Systems.

[11] Lee S., Wong S., Varaiya P. (2017). Group-based hierarchical adaptive traffic-signal control part I: Formulation. Transp. Res. Part B Methodol..

[12] Zeng J., Hu J., Zhang Y. Adaptive traffic signal control with deep recurrent Q-learning. Proceedings of the 2018 IEEE Intelligent Vehicles Symposium (IV).

[13] Liu J., Cai B., Wang J. (2016). Cooperative localization of connected vehicles: Integrating GNSS with DSRC using a robust cubature Kalman filter. IEEE Trans. Intell. Transp. Syst..

[14] Abboud K., Omar H.A., Zhuang W. (2016). Interworking of DSRC and cellular network technologies for V2X communications: A survey. IEEE Trans. Veh. Technol..

[15] Wu X., Subramanian S., Guha R., White R.G., Li J., Lu K.W., Bucceri A., Zhang T. (2013). Vehicular communications using DSRC: Challenges, enhancements, and evolution. IEEE J. Sel. Areas Commun..

[16] Camacho F., Cárdenas C., Muñoz D. (2018). Emerging technologies and research challenges for intelligent transportation systems: 5G, HetNets, and SDN. Int. J. Interact. Des. Manuf..

[17] Wang J., Ni D., Li K. (2014). RFID-based vehicle positioning and its applications in connected vehicles. Sensors.

[18] Tang T., Shi W., Shang H., Wang Y. (2014). A new car-following model with consideration of inter-vehicle communication. Nonlinear Dyn..

[19] Wu W., Head L., Yan S., Ma W. (2018). Development and evaluation of bus lanes with intermittent and dynamic priority in connected vehicle environment. J. Intell. Transp. Syst..

[20] Guo Q., Li L., Ban X.J. (2019). Urban traffic signal control with connected and automated vehicles: A survey. Transp. Res. Part C Emerg. Technol..

[21] Harding J., Powell G., Yoon R., Fikentscher J., Doyle C., Sade D., Lukuc M., Simons J., Wang J. (2014). Vehicle-to-vehicle Communications: Readiness of V2V Technology for Application.

[22] Lozano Domínguez J.M., Mateo Sanguino T.J. (2019). Review on V2X, I2X, and P2X Communications and Their Applications: A Comprehensive Analysis over Time. Sensors.

[23] Omidvar A., Pourmehrab M., Emami P., Kiriazes R., Esposito J.C., Letter C., Elefteriadou L., Crane C.D., Ranka S. (2018). Deployment and testing of optimized autonomous and connected vehicle trajectories at a closed-course signalized intersection. Transp. Res. Rec..

[24] Goli S.A., Far B.H., Fapojuwo A.O. Vehicle Trajectory Prediction with Gaussian Process Regression in Connected Vehicle Environment. Proceedings of the 2018 IEEE Intelligent Vehicles Symposium (IV).

[25] Tiaprasert K., Zhang Y., Wang X.B., Zeng X. (2015). Queue length estimation using connected vehicle technology for adaptive signal control. IEEE Trans. Intell. Transp. Syst..

[26] Li J.-Q., Zhou K., Shladover S.E., Skabardonis A. (2013). Estimating queue length under connected vehicle technology: Using probe vehicle, loop detector, and fused data. Transp. Res. Rec..

[27] Christofa E., Argote J., Skabardonis A. (2013). Arterial queue spillback detection and signal control based on connected vehicle technology. Transp. Res. Rec..

[28] Gao K., Han F., Dong P., Xiong N., Du R. (2019). Connected Vehicle as a Mobile Sensor for Real Time Queue Length at Signalized Intersections. Sensors.

[29] Goodall N.J., Smith B.L., Park B. (2013). Traffic signal control with connected vehicles. Transp. Res. Rec..

[30] He Q., Head K.L., Ding J. (2012). PAMSCOD: Platoon-based arterial multi-modal signal control with online data. Transp. Res. Part C Emerg. Technol..

[31] Wan N., Vahidi A., Luckow A. (2016). Optimal speed advisory for connected vehicles in arterial roads and the impact on mixed traffic. Transp. Res. Part C Emerg. Technol..

[32] Ramezani H., Benekohal R. Optimized speed harmonization with connected vehicles for work zones. Proceedings of the 2015 IEEE 18th International Conference on Intelligent Transportation Systems.

[33] Tajalli M., Hajbabaie A. (2018). Dynamic speed harmonization in connected urban street networks. Comput.-Aided Civ. Infrastruct. Eng..

[34] Wu W., Li P., Zhang Y. (2015). Modelling and simulation of vehicle speed guidance in connected vehicle environment. Int. J. Simul. Model..

[35] Tang T., Zhang J., Liu K. (2017). A speed guidance model accounting for the driver’s bounded rationality at a signalized intersection. Phys. A Stat. Mech. Appl..

[36] Tang T., Yi Z., Zhang J., Wang T., Leng J. (2018). A speed guidance strategy for multiple signalized intersections based on car-following model. Phys. A Stat. Mech. Appl..

[37] Head L., Gettman D., Wei Z. (2006). Decision model for priority control of traffic signals. J. Transp. Res. Rec..

